# Re-evaluating the phylogenetic position of the enigmatic early Cambrian deuterostome *Yanjiahella*

**DOI:** 10.1038/s41467-020-14920-x

**Published:** 2020-03-09

**Authors:** Samuel Zamora, David F. Wright, Rich Mooi, Bertrand Lefebvre, Thomas E. Guensburg, Przemysław Gorzelak, Bruno David, Colin D. Sumrall, Selina R. Cole, Aaron W. Hunter, James Sprinkle, Jeffrey R. Thompson, Timothy A. M. Ewin, Oldřich Fatka, Elise Nardin, Mike Reich, Martina Nohejlová, Imran A. Rahman

**Affiliations:** 1 Instituto Geológico y Minero de España (IGME), 50006 Zaragoza, Spain; 2 Grupo Aragosaurus-IUCA, Área de Paleontología, Facultad de Ciencias, Universidad de Zaragoza, Zaragoza, Spain; 3 Division of Paleontology, American Museum of Natural History, New York, NY 10024 USA; 4 Department of Paleobiology, National Museum of Natural History, Smithsonian Institution, Washington, D.C 20560 USA; 5 Department of Invertebrate Zoology and Geology, California Academy of Sciences, San Francisco, CA 94118-4503 USA; 6 UMR CNRS 5276 LGLTPE, Université Claude-Bernard Lyon 1, Lyon, 69622 France; 7 IRC, Field Museum, Chicago, IL 60605-2496 USA; 8 Institute of Paleobiology, Polish Academy of Sciences, 00-818 Warsaw, Poland; 9 Muséum national d’Histoire naturelle, 75005 Paris, France; 10 Department of Earth and Planetary Sciences, University of Tennessee, Knoxville, TN 37996-15256 USA; 11 Department of Earth Sciences, University of Cambridge, Downing Street, Cambridge, Cambridgeshire CB2 3EQ UK; 12 School of Earth Sciences, The University of Western Australia, Crawley, WA 6009 Australia; 13 Department of Geological Sciences, Jackson School of Geosciences, University of Texas, Austin, TX 78712-0254 USA; 14 University College London, Department of Genetics. Evolution and Environment, London, WC1E 6BT UK; 15 The Natural History Museum London, London, SW7 5BD UK; 16 Department of Geology and Palaeontology, Faculty of Science, Charles University, Praha, 128 43 Czech Republic; 17 Géosciences Environnement Toulouse, Université de Toulouse, CNRS, Toulouse, France; 18 SNSB - Bavarian State Collection of Palaeontology and Geology, 80333 Munich, Germany; 19 Ludwig-Maximilians-Universität München, Department of Earth and Environmental Sciences, Paleontology and Geobiology, 80333 Munich, Germany; 20 GeoBio-CenterLMU, 80333 Munich, Germany; 21 Czech Geological Survey, Prague, 11821 Czech Republic; 22 Oxford University Museum of Natural History, Oxford, OX1 3PW UK

**Keywords:** Palaeontology, Taxonomy

**Arising from** Topper, et al. *Nature Communications* 10.1038/s41467-019-09059-3 (2019)

Echinoderms are a diverse clade of marine invertebrates with a distinctive body plan. Extant forms are characterized by a calcite skeleton with a mesh-like microstructure (stereom), pentaradial symmetry as adults developed after metamorphosis from a bilateral larva, and a water vascular system with tube feet^1^. These characters differentiate echinoderms from their sister group, the bilaterally symmetric hemichordates^2^. The morphological gulf between these phyla means that key questions, such as when and how echinoderms diverged from the latest common ancestor shared with hemichordates, have no immediate answer. Topper et al.^3^ report *Yanjiahella biscarpa* from the Fortunian (~541.0–534.6 Ma) of Hubei Province, China claiming it to be the oldest, most plesiomorphic echinoderm yet discovered. *Yanjiahella* is ~15–20 million years older than the earliest unequivocal echinoderms reported to date^4^. Topper et al.^3^ interpret it as a hemichordate-like echinoderm bridging the gap between these two morphologically disparate sister phyla, with important implications for understanding the origin and early evolution of echinoderms. However, this paper fails to identify a single echinoderm synapomorphy in the fossil material they describe. Moreover, our re-analysis of their phylogenetic character matrix demonstrates that placement of *Yanjiahella* is ambiguous. Affinities of *Yanjiahella* and its potential significance for understanding deuterostome evolution are unclear.

Topper et al.^3^ report no trace of stereom in *Yanjiahella biscarpa*, claiming its absence in the 35 studied specimens results from their preservation as natural molds in gray-black silty-shale, rather than evidence of absence. Most Cambrian echinoderms are preserved as molds, with the original calcite skeleton dissolved during weathering and/or diagenesis. Yet presence of stereom can typically be inferred by direct observations following removal of iron oxides and calcite residues using oxalic acid solutions or latex cast series. Subsequent casting of these molds using latex often reveals relic “ghost” surface textures typical of echinoderms (Fig. 1). Even when these textures are not preserved, latex casting can still provide critical information allowing recognition of skeletal plate arrangements and the position of major apertures. If *Yanjiahella* originally had stereom microstructure in its skeleton, we would expect to see evidence of this in latex casts of the fossils or on the surfaces of the fossils under SEM, once the specimens had been properly prepared, but Topper et al.^3^ carried out no such work. Indeed, many of the morphological uncertainties about *Yanjiahella* expressed in Topper et al.^3^ including the original composition, shape and arrangement of plates, and morphology of the stalk might have been resolved if specimens had been cast in latex for study. Therefore, Topper et al.^3^ omitted a pertinent method to confirm presence of stereom and details of anatomy in their studied specimens.

**Fig. 1:**
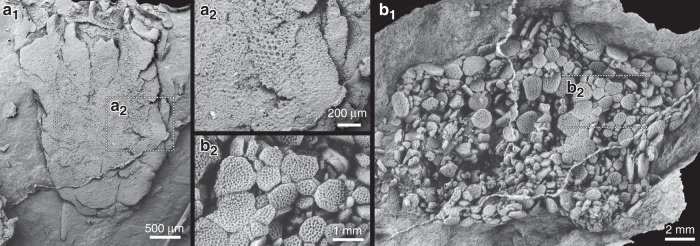
Latex casts of Cambrian echinoderms from the Jbel Wawrmast Formation (Morocco) showing relic “ghost” stereom microstructure associated with the presence of originally calcitic plates. **a** SEM image of a ctenocystoid with a loosely calcified ventral surface. Specimen NHMUK EE15317 (**a**_**1**_). General view (**a**_**2**_). Detail showing stereom in the surface of the plates. **b** Ventral surface of an edrioasteroid. Photograph of latex cast whitened with ammonium chloride sublimate. Specimen NHMUK EE15308 (**b**_**1**_). General view.(**b**_**2**_). Detail of plate sutures and ornamentation. Specimens housed in the Natural History Museum, London (NHMUK).

Additional features raise serious questions about the interpretation of *Yanjiahella* as an echinoderm. In all known early echinoderms, feeding structures take the form of food groves or ambulacra, either embedded in the body wall or erect^1, 5, 6^. These originate at the mouth and are used in feeding. In contrast, in *Yanjiahella*, two putative feeding appendages projected from opposing sides of the thecal margin, with the mouth reconstructed to be between the two appendages. This configuration differs from anything previously reported in echinoderms, including Cambrian forms with paired feeding appendages like *Ubaghsicystis* and *Dibrachicystis*, as well as the two-armed Ordovician echinoderm *Pleurocystites*. Although possessing some plasticity in arm construction, these forms have the same basic design, with flooring plates and cover plates supporting extensions of the water vascular system. That these plate systems have not been described in *Yanjiahella* is problematic, as their demonstrated lack would remove any potential hard part evidence for existence of the water vascular system that forms a crucial synapomorphy for the Echinodermata. Similarly, the stalk of *Yanjiahella* is unlike anything seen in echinoderms. Topper et al.^3^ describe it as consisting of morphologically distinct proximal and distal regions, an arrangement they interpret as novel among echinoderms. This arrangement differs from the posterior appendage in other Cambrian echinoderms, which takes the form of a multi-plated stalk or stem. Furthermore, the apparent bilateral symmetry of *Yanjiahella* is also poor evidence linking the animal with echinoderms because Cambrian representatives of the phylum exhibit a range of symmetries^1^, and all asymmetric and bilateral forms have calcitic skeletons^7^, with at least some showing good evidence of a water vascular system^8^. To conclude, Topper et al.^3^ describe no synapomorphies between *Yanjiahella* and Echinodermata, raising questions as to why it was interpreted as an echinoderm.

To test the findings of Topper et al. independently, we ran their phylogenetic analysis using both the methods described in the paper and additional model-based approaches accounting for differences in rates of character change. Regardless of methods or software implementation, we could not reproduce the Topper et al.^3^ topology (fig. 3, 15 trees of length 71). We infer shorter parsimony trees than those given in the main text of their paper and our strict consensus does not unambiguously place *Yanjiahella* with either echinoderms or hemichordates (Fig. 2a). Under standard assumptions of parsimony, Topper et al.’s^3^ fig. 3 must be rejected for the one presented here (Fig. 2a). Moreover, the 50% majority rule parsimony tree places *Yanjiahella* with hemichordates (Fig. 2b). Indeed, while it is fully acknowledged in the Supplementary Information that results were ambiguous (Topper et al.^3^: Supplementary fig. 7), a less optimal tree is depicted in the main text.

**Fig. 2:**
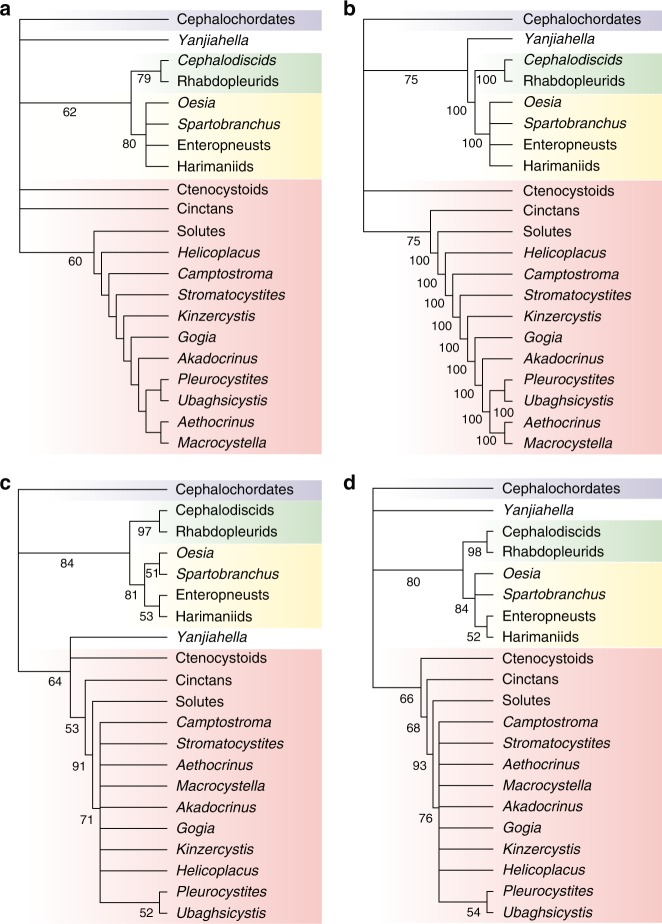
Phylogenetic uncertainty in placement of *Yanjiahella*. **a** Strict consensus of twelve most parsimonious trees (MPTs) recovered via maximum parsimony analysis of the matrix in Topper et al.^3^ (including parsimony uninformative characters: tree length = 70, CI = 0.657; excluding parsimony uninformative characters: tree length = 63, CI = 0.619), node values = bootstrap support via 1,000 replicates. **b** parsimony-based 50% majority rule tree, node values = clade frequencies across MPTs. **c** Bayesian 50% majority rule tree of the matrix in Topper et al.^3^, node values = posterior probability (%). **d** Bayesian 50% majority rule tree from a matrix in which *Yanjiahella* is re-scored as lacking stereom (character 16), node values = posterior probability (%).

Bayesian phylogenetic analysis places *Yanjiahella* as a stem echinoderm (Fig. 2c), but ancestral state reconstruction reveals only one character in the matrix unambiguously supporting its position: the presence of plate-like ossicles embedded in the body wall (character 15). However, this character may be misleading because similarly embedded plate-like ossicles have been observed in other Cambrian animals originally described as echinoderms, including the stem entoproct *Cotyledion tylodes*^9^. Many characters described for *Yanjiahella* by Topper et al.^3^ are either plesiomorphic or otherwise not diagnostic. The two feeding appendages of *Yanjiahella* are similar to the paired tentacles observed in *Herpetogaster collinsi*^10^, while the ridges in the proximal stalk are similar in appearance to the gill bars described in the enteropneust hemichordate *Oesia disjuncta*^11^. Thus, we find no clear synapomorphies uniting *Yanjiahella* with echinoderms.

To illustrate further both the sensitivity in *Yanjiahella*’s phylogenetic position and the importance of using appropriate methods to study echinoderm morphology, we conducted a “thought experiment” analysis in which the presence of stereom in *Yanjiahella* was re-scored from ambiguous to absent (see Methods). If we follow Topper et al.^3^ in assuming cephalochordates as the outgroup, our results indicate this conservative change to the matrix results in equivocal support for *Yanjiahella* as either a stem echinoderm, stem hemichordate, or stem ambulacrarian (Fig. 2d).

Recognizing stem echinoderms in the fossil record is key to uncovering the origin and early evolution of the phylum^1^. If Topper et al.^3^ are correct in interpreting *Yanjiahella* as the most plesiomorphic stem echinoderm, then this would be a highly significant discovery for echinoderm paleobiology. Even if *Yanjiahella* is not vindicated as a stem echinoderm, it is clearly an important and intriguing fossil that could help throw light on other aspects of early deuterostome evolution. In our view, further work is needed to determine accurately its morphology and phylogenetic placement.

## Methods

### Character matrices

To quantify support for the phylogenetic placement of *Yanjiahella*, we re-analyzed the original matrix in the Supplemental Information of Topper et al.^3^ using both maximum parsimony and Bayesian approaches. In contrast to Topper et al.^3^ (cf. fig. 3 and Supplementary figs. 6, 7), we present results matching each method’s respective optimality criteria (e.g., minimum-length trees for parsimony analysis) only. We also conducted analyses with *Yanjiahella* re-scored as lacking stereom (i.e., changing character 16 from “?” to “0”). We note that 7 of the 42 characters (~17%) used by Topper et al.^3^ are not parsimony informative.

### Phylogenetic analyses

Parsimony analyses were conducted in PAUP* 4.0a^12^ using a heuristic search of 1,000 random addition sequence replicates with branch swapping via tree-bisection-reconnection. Branches with a minimum length of zero were collapsed. All characters were unordered and equally weighted. Bootstrap support was evaluated across 10,000 replicate matrices of the 35 parsimony-informative characters included in Topper et al.^3^. Summary statistics for the set of most parsimonious trees (MPTs) were calculated in PAUP* for both the total set of 42 characters and 35 parsimony-informative characters. To further compare our results with Topper et al.’s^3^ figure 3, we also conducted analyses in TNT^13^ using implicit enumeration with branch collapse settings off. Bayesian analyses were conducted in MrBayes 3.2.5^14^ using Markov chain Monte-Carlo (MCMC). We used the standard Mk model of morphological evolution with gamma distributed rate variation and a compound Dirichlet prior on branch lengths. Two runs of four MCMC chains were sampled every 5000 generations across 5 million generations, discarding the first 25% as burn-in. MCMC convergence was assessed using standard MrBayes diagnostics, including the standard deviation of clade frequencies and potential scale reduction factor^14^. Clade support was evaluated using posterior probabilities for nodes retained in the 50% majority rule consensus tree. Ancestral state reconstructions were conducted in Mesquite^15^. A batch file containing the data and scripts to reproduce our phylogenetic results is provided in Supplementary Data 1.

### Reporting summary

Further information on research design is available in the Nature Research Reporting Summary linked to this article.

## Supplementary information

Reporting Summary

Description of Additional Supplementary Files

Supplementary Data 1

## Data Availability

The authors declare that all data supporting the findings of this study are available within the paper and its supplementary information.

## References

[1] Zamora S, Rahman IA (2014). Deciphering the early evolution of echinoderms with Cambrian fossils. Palaeontology.

[2] Cannon JT (2014). Phylogenomic resolution of the hemichordate and echinoderm clade. Curr. Biol..

[3] Topper TP (2019). A stem group echinoderm from the basal Cambrian of China and the origins of Ambulacraria. Nat. Commun..

[4] Zamora S (2013). Cambrian echinoderm diversity and palaeobiogeography. Geol. Soc. Lon. Mem..

[5] Rahman IA, Clausen S (2009). Re-evaluating the palaeobiology and affinities of the Ctenocystoidea (Echinodermata). J. Syst. Palaeont.

[6] Rahman IA, Zamora S (2009). The oldest cinctan carpoid (stem-group Echinodermata), and the evolution of the water vascular system. Zool. J. Linn. Soc..

[7] Zamora S (2012). Plated Cambrian bilaterians reveal the earliest stages of echinoderm evolution. PLoS ONE.

[8] Lefebvre B (2019). Exceptionally preserved soft parts in fossils from the Lower Ordovician of Morocco clarify stylophoran affinities within basal deuterostomes. Geobios.

[9] Zhang Z (2013). A sclerite-bearing stem group entoproct from the early Cambrian and its implications. Sci. Rep..

[10] Caron JB (2010). Tentaculate fossils from the Cambrian of Canada (British Columbia) and China (Yunnan) interpreted as primitive deuterostomes. PLoS ONE.

[11] Nanglu K (2016). Cambrian suspension-feeding tubicolous hemichordates. BMC Biol..

[12] Swofford, D. L. *PAUP*. Phylogenetic Analysis Using Parsimony (*and Other Methods)*. Version 4.0a. (Sinauer Associates, Sunderland, Mass., 2003).

[13] Goloboff PA (2008). TNT, a free program for phylogenetic analysis. Cladistics.

[14] Ronquist F (2012). MrBayes 3.2: efficient Bayesian phylogenetic inference and model choice across a large model space. Syst. Biol..

[15] Maddison, W. P. & Maddison, D. R. *Mesquite: A Modular System for Evolutionary Analysis. Version 3.51* (www.mesquiteproject.org, 2018).

