# Supplementary material for: Re-evaluating the phylogenetic position of the enigmatic early Cambrian deuterostome Yanjiahella
Source: Nat Commun. 2020 Mar 9;11:1286. doi: 10.1038/s41467-020-14920-x (PMC7063041; doi:10.1038/s41467-020-14920-x)
Supplement: Supplementary file 2 — Description of Additional Supplementary Files [file 41467_2020_14920_MOESM2_ESM.pdf]

Description of Additional Supplementary Files

File Name: Supplementary Data 1
Description: Batch file containing the data and scripts to run the analyses.
